# Exploring the Effect of Gastrointestinal *Prevotella* on Growth Performance Traits in Livestock Animals

**DOI:** 10.3390/ani14131965

**Published:** 2024-07-02

**Authors:** Xiyan Kou, Qingshan Ma, Yihong Liu, Muhammad Zahoor Khan, Boxian Wu, Wenting Chen, Xiaotong Liu, Changfa Wang, Yan Li

**Affiliations:** 1 School of Agricultural Science and Engineering, Liaocheng University, Liaocheng 252000, China; 2 Shandong Dong’e Black Donkey Husbandry Technology Co., Ltd., Liaocheng 252000, China

**Keywords:** *Prevotella*, livestock, growth performance, mechanism, application

## Abstract

**Simple Summary:**

*Prevotella* is a strictly anaerobic bacterium that is commonly found in the gastrointestinal tract of domestic animals. This paper presents a thorough review of the current research on the functions, potential mechanisms, and applications of *Prevotella* within the gastrointestinal system. The findings suggest that *Prevotella* plays a crucial role in the metabolism of carbohydrates, lipids, and amino acids in the host. Additionally, there is a notable correlation between the presence of *Prevotella* and the growth performance of livestock. Future research and practical applications aim to delve further into the potential of *Prevotella*, contributing to the sustainable development of the livestock industry.

**Abstract:**

Microorganisms in the rumen play a crucial role in determining the most efficient utilization rate of nutrients. Among these microorganisms, *Prevotella* stands out as one of the most representative bacteria within the rumen biological system. *Prevotella* is a common strict anaerobic bacterium that is found in the gastrointestinal tract of livestock. *Prevotella* plays a crucial role in breaking down and metabolizing complex nutrients like cellulose and protein during food digestion. Moreover, it is capable of working together with other bacteria in the body’s digestive system. Several studies have shown a strong correlation between the abundance of *Prevotella* and livestock growth performance. This paper provides a comprehensive review of the current research on the function, mechanisms, and applications of *Prevotella* in the gastrointestinal tract. The insights provided in this review could serve as a theoretical basis for accurately classifying *Prevotella,* further investigating its effects and potential mechanisms on livestock growth performance, and exploring its practical applications.

## 1. Introduction

The intestinal microbiota is a complex ecosystem comprising diverse microbial communities such as bacteria, anaerobic fungi, methanogenic archaea, and ciliate protozoa, which coexist symbiotically with their host organisms [1,2]. Intestinal microbiota and their hosts have developed a complex and mutually adapted micro-ecological system [3]. This steady microbiome-host balance is essential for the maintenance and ideal physiological function of the intestine [4]. Consistently, studies have shown that these microbial communities are known to influence various aspects of host physiology, such as nutrient metabolism, immune system function, and overall health [5,6]. Therefore, many factors influence the diversity and function of gut microbes, such as environmental factors, diet, antibiotic use, lifestyle, genetics, age, and so on [7,8,9,10,11,12,13,14].

The associations of gut microbiota with health and production performance have been extensively studied in livestock animals including donkeys [15,16,17,18], horses [19,20,21,22,23], cattle [24,25,26,27,28,29], buffalo [30,31,32], pigs [33,34], sheep and goats [5,35,36,37,38,39,40,41,42]. The growth performance of livestock is directly related to the economic benefits of farming. The degree of digestion and absorption of nutrients directly affects the growth performance of the livestock. The intestinal tract is a key site for nutrient digestion and absorption in animals, with a large number of microorganisms living in symbiosis. Studies have shown that intestinal microorganisms play an extremely important role in maintaining the normal functioning of the intestinal tract, influencing the host’s growth performance [43]. With the development of high-throughput sequencing and anaerobic pure culture techniques, the roles of strict anaerobes in the intestinal tract, such as Bifidobacterium and *Prevotella*, are gradually being revealed. Among them, *Prevotella* is commonly found in the intestinal tracts of a wide range of animals, including humans [44], mice [45], ruminants [46], and poultry [47], where it contributes significantly to carbohydrate, lipid, and amino acid metabolism [48,49,50,51] and is associated with growth performance. The populations of *Prevotella ruminicola* were enhanced by increasing guanidinoacetic acid (GAA) addition [52] and were associated with feed efficiency [53]. Furthermore, some *Prevotella* species may be potentially infectious agents for livestock; one study found that *Prevotella melaninogenica* is a causative agent of endometritis in cattle [29]. Interestingly, *Prevotella*, a representative genus of Bacteroidetes, was also a key microbe that was shown to be useful in improving disease prognosis [54]. Consistently, an increased abundance of *Prevotella* has been found to be correlated with several pathways related to amino acid and carbohydrate metabolism pathways, including branched-chain amino acid metabolism [55]. Additionally, *Prevotella ruminicola* has been shown to play a crucial role in the proteolysis of cereal grains [56]. Considering the significance of *Prevotella*, this review paper aims to outline the recent research reports on the classification, characteristics, distribution, and correlation between *Prevotella* in the gastrointestinal tract and growth performance. The objective is to provide a theoretical foundation for further investigations into the role of *Prevotella* and the identification of new probiotic strains.

## 2. Animal Microbiota and Their Role in Livestock Animals Growth Performance

The microbiome plays a crucial role in the ecology and metabolic abilities of many animals, making it vital for their survival and persistence over time and space. One area where microbial gut communities are especially significant is in their mutualistic symbiotic relationships with the host. These communities contribute to various important functions for the host, including digestion, immunity, and even behavior [57,58,59,60,61]. Consistently, the direct relationship of gut microbiota with growth performance has been documented in several livestock animals [62] including pig [63,64,65,66,67], cattle [68,69,70,71], sheep [39,72,73,74]; goats [35,75,76], and donkeys [15,77,78]. While the microbiota plays an important role in maintaining livestock health and improving growth performance, the microbiota can be affected by various factors such as dietary changes, antibiotics, and probiotic supplements. Therefore, the discovery of animal microbiota and their role in livestock growth performance, and the identification of potential associations between microbiota and livestock growth performance will provide important information for the development of intervention strategies aimed at improving livestock growth.

## 3. Gastrointestinal *Prevotella*

### 3.1. Brief Description of Prevotella

*Prevotella* belongs to the Bacteroidetes, Bacteroidia, Bacteroidales, and Prevotellaceae. The *Prevotella* genus was created in 1990 by Shah and Collins after reclassification from the genus *Mycobacterium* [79]. Following the naming of the new genus *Prevotella* by French microbiologist A.R. Prevot, 16 species of Gram-negative, specialized anaerobes were reclassified, eventually to the genus *Prevotella*. *Prevotella* spp. are polymorphic bacilli, non-spore-producing, Gram-negative organisms, which are very demanding on growth conditions, requiring a strictly anaerobic environment, with an optimal growth temperature of 37 ℃, and their growth can be inhibited by 20% (*w*/*v*) bile acid or 6.5% NaCl. The main fermentation products of *Prevotella* are acetic acid, succinic acid, and small amounts of isobutyric, isovaleric, and lactic acids. Currently, *Prevotella* can be isolated from a variety of environments, such as the human oral cavity, skin abscesses, intestinal contents, and soil. *Prevotella* is also prevalent in the rumen and the intestinal tract of monogastric, where it affects growth performance [80].

### 3.2. Classification of the Prevotella

**Figure 1:**
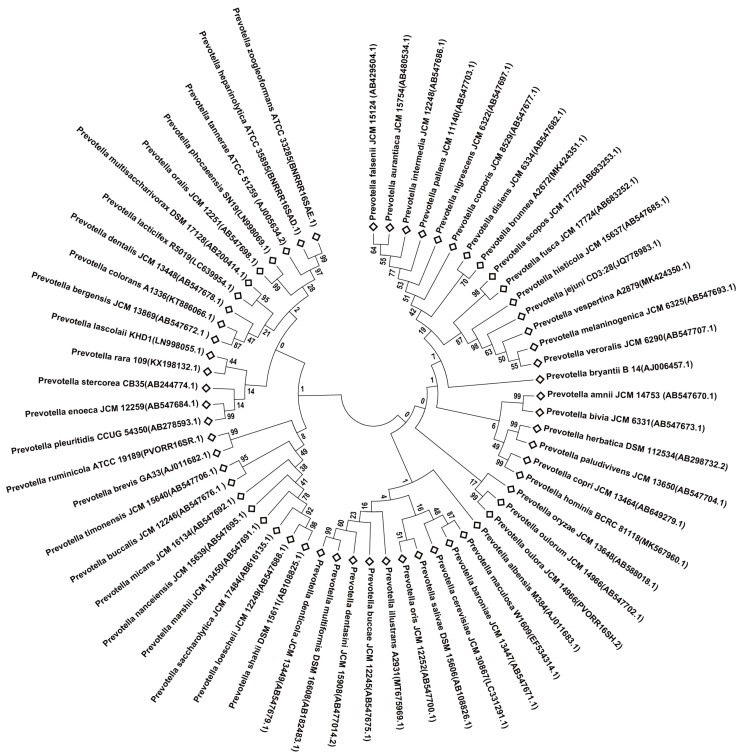
Phylogenetic tree based on 16S rRNA gene sequence of *Prevotella* spp., The tree was constructed with MEGA7 by the neighbor-joining (NJ) method with 1000 bootstrap replicates. Branches corresponding to partitions reproduced in less than 50% of bootstrap replicates were collapsed.

Currently, the genus *Prevotella* officially published in the List of Prokaryotic names with Standing in Nomenclature (LPSN) consists of 62 species and one subspecies. The phylogenetic tree was constructed based on the 16S rRNA sequences of these *Prevotella* species (Figure 1), and all of them, except for *Prevotella mizrahii*, have been subjected to whole-genome sequencing, with the genome sizes ranging from 1413 bp to 1535 bp. The reclassification of *Prevotella* has been discussed, but no official classification basis has been published. Avguštin et al. [81] proposed to compare “rumen” and “non-rumen” *Prevotella* separately, and in genome analyses of *Prevotella*, taxonomic comparisons have also begun to be made between “ruminant” and “human isolates”. The basis for the classification of *Prevotella* has evolved with advances in technology. Early classification was based on 16S rRNA gene sequence comparisons, but as more and more *Prevotella* were discovered, 16S rRNA gene sequence comparisons alone did not provide sufficient evidence to accurately differentiate the *Prevotella*. In this regard, Hitch et al. [82] have reassessed the relevance of species within the genus *Prevotella* based on a multi-genomic approach; by building a phylogenetic tree using three different sets of marker genes, performing average amino acid identity (AAI) and percentage of conserved proteins (POCP) comparisons, pan-genomic identification of specific marker genes, protein families (Pfam) and carbohydrate-activating enzyme (CAZyme) with 19,000 amplicon samples were analyzed to show the metabolic profiles of different *Prevotella* species for precise strain identification.

### 3.3. Characteristics of the Distribution of Prevotella in the Gastrointestinal Tract

The species, number, and distribution of *Prevotella* can vary due to differences in gastrointestinal tract structures, internal environments, and growth stages among animals. *Prevotella* richness gradually increases from the proximal to distal gastrointestinal tract with increasing pH [83,84]. *Prevotella* is present throughout the gastrointestinal tract of animals. Xie et al. [85] found that *Prevotella* was more abundant in the stomach than in the intestine and was highest in the rumen of seven ruminant species (cow, buffalo, yak, goat, sheep, deer, and roe deer). Consistently, Li et al. [86] found that in the gastrointestinal tract of donkeys, the cecum was more abundant in *Prevotella*. Not only the gut, but also the mucosa is an important site for *Prevotella* parasitism, and Looft et al. [87] observed that in the ileum of adult pigs, the concentration of *Prevotella* in the intestinal mucosal microbiota was significantly higher than in the intestinal microbiota. Also, *Prevotella* load fluctuates with the growth stage. Studies have shown that *Prevotella gastrointestinalis* is less abundant during lactation but becomes the dominant flora during the growth and fattening stages after weaning [88,89,90,91,92]. This is equally supported by another study in calves [93], where the rumen microflora was dominated by Bacteroidetes during the first month of life, with *Prevotella* becoming the dominant family as they aged.

The species, number, and distribution of *Prevotella* are also influenced by what is going on in the intestinal environment. Wang et al. [88] found *Prevotella* as a core group of growing bacteria that can interact with other microbial community members. Studies in pigs have shown that *Prevotella* can co-operate with Lactobacillus in the intestinal tract to break down a wide range of plant polysaccharides in order to adapt to changes in dietary conditions after weaning [90]. Fehlner et al. [94] found that mutual co-operation between different *Prevotella* species was able to catabolize richer and more complex polysaccharides. Interactions between other microorganisms in the intestinal environment and *Prevotella* may be an important factor influencing the behavior of *Prevotella* in different intestinal ecosystems and its interactions with the host. Liang et al. [95] found that during digestion of corn stover by Angus bull, *Prevotella* may synergize with other fungi (*Ascomycota*, *Basidioycota*, *Mucoromycota,* and *Chytridiomycota*) to degrade cellulose.

It can be seen that differences in the structure of the gastrointestinal tract, the internal environment, microbial interactions in the gastrointestinal tract, and the stage of growth of animals affect the species and abundance of *Prevotella* and further influence metabolic processes such as the breakdown of complex carbohydrates in the gastrointestinal tract, which may have an impact on livestock growth performance.

## 4. Effect of *Prevotella* on Growth Performance Traits of Livestock Animals

**Figure 2:**
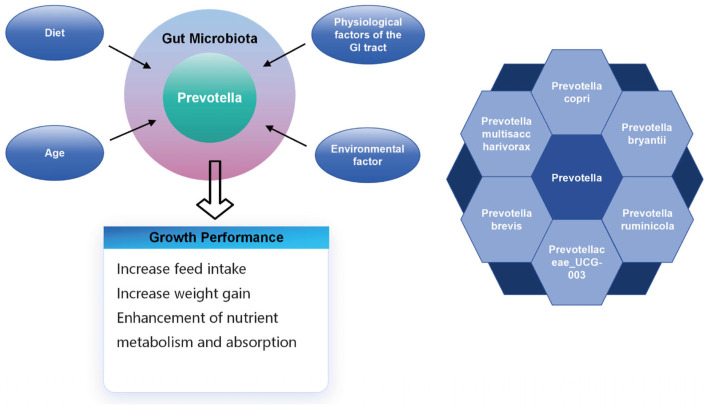
Schematic representation of factors influencing the gastrointestinal microbiota of domestic animals, and the hypothesized role of *Prevotella* in regulating livestock growth performance traits including weight gain, increased feed intake, and metabolism. Note: GI stands for gastrointestinal.

Gastrointestinal *Prevotella* can be influenced by various factors such as diet [64], age, gastrointestinal physiological factors, and environmental factors. The complex of influences triggers changes in the biomass of different *Prevotella* species, which in turn improves the growth performance of livestock (Figure 2). Mechanism-wise, they revealed that *Prevotella* can enhance the secretion of enzymes responsible for nutrition degradation and increase the production of propionate, potentially impacting the gluconeogenesis pathway to provide more energy for the host [96]. Another study in rats indicated that *Prevotella* may regulate feed intake through the gut–brain axis by influencing serum ghrelin levels [97]. However, further research is required to fully elucidate the precise mechanism.

### 4.1. Feeding Strategies to Improve Gastrointestinal Prevotella in Ruminant Livestock and Their Association with Growth Performance Traits

*Prevotella* is one of the richest flora in the gastrointestinal tract of livestock and is identified mainly in the rumen. Gastrointestinal *Prevotella* in rumen livestock can be affected by feeding strategies that affect livestock growth performance. Cui et al. [98] found that changing the feeding strategy of beef cattle to whole-plant corn silage improved the level of Prevotellaceae_UCG-003, significantly increased daily gain, and decreased the feed intake-to-weight gain ratio of beef cattle. This facilitates positive regulation of several beneficial biological processes including amino acid metabolism, carbohydrate metabolism, energy metabolism, genetic information processing, lipid metabolism, membrane transport, metabolism of cofactors and vitamins, nucleotide metabolism, replication and repair, and translation. In studies with beef cattle, it has been found that the addition of different ingredients to the ration improves different *Prevotella* strains to enhance growth performance. For example, the addition of lactic acid bacteria probiotics to the ration improves *Prevotella copri* and *Prevotella stercorea* and helps the host to digest complex carbohydrates such as starch [99]; additional addition of yeast and yeast cell wall polysaccharides can improve *Prevotella brevis* B14; *Prevotella bryantii* GA33; and *Prevotella ruminicola* 23 with an increase in mean daily weight gain and improve growth performance [100]. In a study where different tropical agricultural by-products were fed to buffaloes in place of conventional feeds, complete replacement of regular feed concentrates with palm kernel cake and corn gluten was found to result in increased load of *Prevotella* in the rumen, lower average daily gain and digestibility, and reduced growth performance, whereas the use of cassava residue and dried distiller’s grain for alternative feeding affects the reduction in *Prevotella* concentration, but resulted in a significant increase in average daily gain and an increase in growth performance [101]. Similar findings have been made not only in cattle and buffalo but also in sheep, where Wu et al. [96] found that the addition of cysteamine to lambs’ rations enhanced *Prevotella* to enhance growth performance and rumen fermentation. Zhang et al. [102] conducted a study in which they substituted 33% of corn starch with barley starch in the diet of sheep. They found that this substitution improved *Prevotella brevis*, enhanced the rumen environment, and increased feed efficiency. Rehemujiang et al. [103] discovered that feeding sheep a fermented total mixed ration containing cottonseed meal resulted in improved *Prevotella* levels, increased volatile fatty acid production, promoted average daily weight gain, and enhanced overall performance. In a study by Yu et al. [42], it was found that thymol supplementation reduced the presence of *Prevotella melaninogenica* and *Prevotella ruminicola* in the rumen of goats. *P. melaninogenica* is associated with xylitol fermentation, while *P. ruminicola* is involved in cellulose and pectin degradation, as well as protein and peptide metabolism. Thus, reductions in *P. melaninogenica* and *P. ruminicola* may be detrimental to the breakdown of proteins and fibrous material, resulting in reduced methane production. In addition to modifying the composition of the ration, optimizing feeding patterns can also influence the prevalence of *Prevotella*, thereby enhancing the overall performance of livestock. Wang et al. [104] found that rearing calves through the concentrate plus hay feeding pattern could increase the content of *Prevotella multisaccharivorax*, which is more helpful to the digestion of carbohydrates and alleviates inflammation. Simultaneously, the synergistic effect with the host could hydrolyze lipid substances more efficiently and promote the absorption of fat by the organism. In a study on Tibetan sheep, it was found that indoor feeding regimes could regulate amino acids, lipid, and carbohydrate metabolism in muscle tissues and improve meat quality by increasing *Prevotella* [105].

### 4.2. Feeding Strategies to Improve Gastrointestinal Prevotella and Their Effect on Growth Performance Traits in Donkeys and Pigs

Huang et al. [15] found that the addition of yeast polysaccharide to the ration improved the metabolic mechanisms by which *Prevotella* promotes growth-promoting and immune-regulating effects. Donkeys with corn straw diets were fed ad libitum in addition to a commercial concentrate diet also improves *Prevotella* and affects lipid metabolism and immune responses [86]. In studies on pigs, the addition of tryptophan and glutamic acid to diets can improve intestinal health and growth performance by increasing *Prevotella* load to stabilize the intestinal environment and immune status [34]. Hu et al. [106] added vanillic acid to piglet diets and found that it could improve *Prevotella* and increase the final body weight and average daily gain.

Different feeding strategies cause improvements in different *Prevotella* species, resulting in complex results (Table 1). This phenomenon may be attributed to the notable diversity observed among *Prevotella* in their capacity to utilize complex polysaccharides [107], or to the significant discrepancies in the role played by *Prevotella* between ruminant and non-ruminant livestock. Further in-depth studies utilizing pure cultures, sterile animals, and genomics techniques are required to accurately investigate the roles of specific strains of *Prevotella* on livestock growth and development, and thus to explore their causality in altering livestock growth. Furthermore, the causal role of specific *Prevotella* strains in altering the growth performance of livestock must be investigated.

## 5. Involvement of *Prevotella* in Metabolism of Livestock Animals

*Prevotella* has an important contribution to carbohydrate metabolism, lipid metabolism, and amino acid metabolism in livestock, which affects growth performance.

### 5.1. Involvement of Prevotella in Carbohydrate Metabolism

As an important source of nutrition for most livestock, the ability of the animal body to digest and absorb carbohydrates will directly affect the growth performance of the organism. *Prevotella* is abundantly distributed in the rumen of ruminants or the gastrointestinal tract of other herbivores and can degrade and utilize a wide range of polysaccharides [80], from which it derives energy to sustain the growth of the animal. There are differences in the polysaccharides that can be metabolized by different *Prevotella* species. Peach et al. [94] found that *Prevotella* has Polysaccharide Utilization Loci (PULs) encoding a variety of carbohydrate-active enzymes by macro-genome sequencing techniques, *P. copri* can degrade a wide range of hemicelluloses in the plant such as xylan, arabinose, mannose, etc., whereas *P. stercorea* has only a low ability to metabolize hemicellulose. It can be seen that there are large differences in complex polysaccharide degradation by different *Prevotella* species, which may be one of the reasons for their different correlations with livestock growth performance. In addition, the synergistic interactions of different *Prevotella* species play an important role in the degradation of complex carbohydrates in the gastrointestinal tract. It was found that *P. stercorea* has uncommon carbohydrate esterases compared to *P. copri* and also has a wide range of amino acid metabolism functions; this led to the speculation that *P. stercorea* may act to remove ester group modifications from complex carbohydrates in order to promote sugar hydrolysis [108].

### 5.2. Involvement of Prevotella in the Regulation of Host Lipid Metabolism

**Figure 3:**
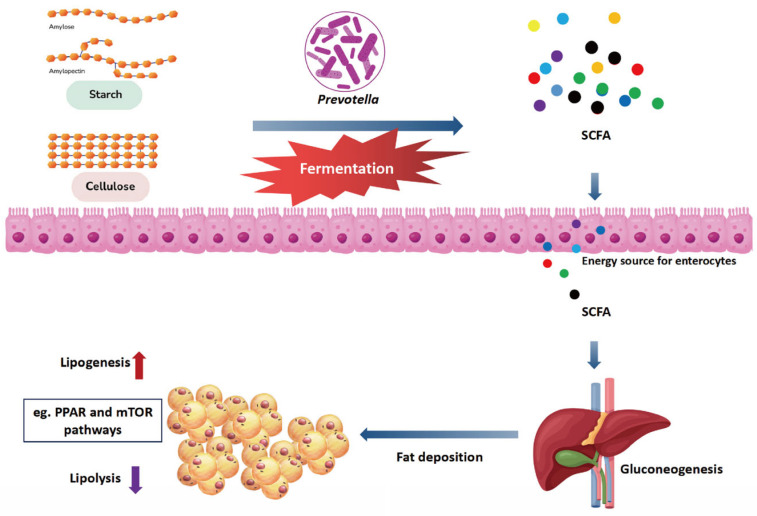
Possible mechanisms of *Prevotella* that regulate host fat deposition. SCFA, Short-chain fatty acids.

In human studies, the concentration of *Prevotella* or the ratio of *Prevotella* to *Anthrobacter* have been identified as key biomarkers for adiposity loss and weight management [109,110]. Similarly, studies in livestock have demonstrated that alterations in Prevotella levels influence fat deposition [111,112]. In a comparable study, Conte et al. [113] reported a positive correlation between *Prevotella* richness and lipid metabolism in dairy cows. In yaks, *Prevotella* in the rumen was positively and negatively correlated with polyunsaturated and saturated fatty acids, respectively [114]. Interestingly *Prevotella intestinalis* also showed significantly higher mean daily feed intake and growth performance than other intestinal types [112,115]. In terms of possible mechanisms, a previous study by our group [86] showed that donkey intestinal *Prevotella* load and intestinal lipid metabolism genes (e.g., *PPARγ*, *ME1*, *MBOAT1*, *ACOX1*, *ACOX2*, *LIPH*) are positively correlated or regulate host lipid metabolism through signaling pathways associated with these genes. A review of the available research reports (Figure 3) suggests that the possible mechanism is that *Prevotella* can efficiently derive energy from complex polysaccharides [116] through fermentation of soluble carbohydrates and degradation of insoluble plant fibers to produce short-chain fatty acids, which can be used as substrates for lipogenesis, gluconeogenesis, and cholesterol synthesis [43], which in turn can increase the fat deposition in the host. It has also been found that *Prevotella* can elevate the abundance of lipopolysaccharides and branched-chain amino acids in serum, which activates the mTOR signaling pathway, promotes liposynthesis, and reduces lipolysis, thus further increasing the level of fat deposition in the host [117]. Although existing studies have found that *Prevotella* can enhance host fat deposition, the specific regulatory pathway is still not very clear, coupled with the fact that different *Prevotella* strains are functionally different, and isolation and pure culture of *Prevotella* strains are also very difficult, so it is still difficult to explore the mechanism by which *Prevotella* regulates host fat metabolism in the gastrointestinal tract of livestock.

### 5.3. Involvement of Prevotella in Host Amino Acid Metabolism

Amino acid metabolism, as one of the three major substance metabolisms, plays a crucial role in maintaining metabolic homeostasis in the body [118]. It was found that *Prevotella* is often positively correlated with amino acid metabolism [119]. Xue et al. [120] found that *Prevotella* may be involved in host amino acid metabolism including glycine, serine, threonine, alanine, aspartic acid, glutamic acid, cysteine, and methionine by using multi-omics techniques. Takahashi et al. [51] further investigated and confirmed the pathway of *Prevotella* intermedia and *Prevotella nigrescens* strains to metabolize aspartic acid, valine, and leucine with metabolic end products such as succinic acid, acetic acid, isobutyric acid, isovaleric acid. Among them, succinic acid and acetic acid can enter the tricarboxylic acid cycle to participate in the growth and metabolism of the organism, and isobutyric acid and isovaleric acid can be used as precursors of short-chain fatty acids [118]. *Prevotella* can release ammonia by amino acid deamination and urease hydrolysis of urea, which can later be used for metabolism and protein synthesis [121].

## 6. Prospects for the Application of *Prevotella*

Currently, research on the application of *Prevotella* has been carried out in various areas. In animal husbandry, *Prevotella* has the potential to be used as an antimethanogenic agent. One of the end products of rumen fermentation in ruminants is methane, which is a polluting greenhouse gas and can characterize the energy loss of livestock [122,123,124,125]. Thus, enabling livestock to reduce methane production is beneficial for energy utilization and environmental protection. It has been found that *Prevotella* can sequester hydrogen by fermenting sugar or lactic acid via the succinate or acrylic acid pathways, respectively [126,127], to reduce methane production. Roehe et al. [128] found an abundance and high density of *Prevotella* in Holstein cattle herds that produced less methane under the same feeding conditions. Thus, *Prevotella* can be used as an applied research direction to reduce methane production. *Prevotella* can also be used as a probiotic to improve livestock growth performance. Probiotics are important potential alternatives to antibiotic growth promoters [129], not only to improve digestion and absorption but also to influence the immune and antioxidant functions of livestock. Currently, *Prevotella* may be used as a probiotic in early lactation dairy cows and sheep to improve rumen fermentation products and milk fat concentration and may be developed as a feed additive to improve feed efficiency [130,131,132]. However, the application of *Prevotella* is not widespread at the moment, a wide range of practical applications can be developed on the basis of theoretical research. For example, the development of functional probiotics, through the combination of genetic recombination, transfection, and other genetic engineering techniques, to improve strain resistance and effective expression of beneficial metabolites, to accelerate the research of symbiotic factors, the development of complex functional probiotics, and so on.

## 7. Conclusions

In recent years, significant progress has been made in studying the influence of the gastrointestinal microbiota on livestock growth performance. However, research in this area is still limited to examining the association between overall community diversity and host phenotype. Therefore, it is crucial to determine the causal effects of individual members of the gastrointestinal microbiota on host phenotypes in order to improve livestock growth performance and productivity. For instance, *Prevotella* has been found to have varying effects on the host in the porcine gastrointestinal tract. This suggests that it is necessary to clearly classify individual strains of the *Prevotella* genus and conduct studies that are specific to the genus or even individual strains before investigating their associated effects. The increasing use of technologies such as genomics, proteomics, and metabolomics in functional studies of the microbiota allows for a comprehensive characterization of the composition, function, and metabolic activity of complex microbiota, even at the strain level. With the declining cost of multi-omics analyses and the rapid development of data integration, multi-omics technologies will be increasingly employed in studying the gastrointestinal microbiota.

Future research is expected to focus on screening the pure culture of *Prevotella gastrointestinalis*, constructing a whole genome reference database for *Prevotella,* improving the accuracy of gene annotation, further analyzing the function of the genus and even the microbial community through multi-omics analysis technology, and elucidating the cause-and-effect relationship between phenotypes and the mechanism of action. Based on these findings, practical applications of *Prevotella* can be developed in multiple directions.

## Figures and Tables

**Table 1:** Feeding strategies to improve gastrointestinal *Prevotella* in livestock and their relationship with growth performance traits.

Feed Type	Effect on Microbiota	Outcomes	Animal Species	References
Whole-plant corn silage	Improved the Prevotellaceae_UCG-003	✧ Positively regulated several beneficial biological processes including amino acid metabolism, carbohydrate metabolism, energy metabolism, genetic information processing, lipid metabolism, membrane transport, metabolism of cofactors and vitamins, nucleotide metabolism, replication and repair, and translation; Significantly increased daily gain and decreased the feed intake-to-weight gain ratio	Beef cattle	[98]
Yeast and yeast cell wall polysaccharides	*Prevotella brevis* B1 4; *Prevotella bryantii* GA33; *Prevotella ruminicola* 23	✧ Increased average daily weight gain, improved growth performance	Beef cattle	[100]
Cysteamine	*Prevotella*	✧ Enhanced growth performance and rumen fermentation	Lambs	[96]
Lactic acid bacteria probiotics	Improved the *Prevotella copri*; *Prevotella stercorea*	✧ Aid the host in the digestion of complex carbohydrates such as starch	Cattle	[99]
Concentrate plus hay feeding pattern	*Prevotella multisaccharivorax*	✧ More helpful to the digestion of carbohydrates and alleviated inflammation. Simultaneously, the synergistic effect with the host could hydrolyze lipid substances more efficiently and promote the absorption of fat by the organism.	Calves	[104]
Lyophilized probiotic formulation (*Limosilactobacillus reuteri* BF-E7 and *Ligilactobacillus salivarius* BF-17)	Increased concentration of *Prevotella*	✧ Significant increase in final body weight, average daily weight gain, average dry matter intake, and structural growth indicators	Buffalo calves	[31]
Ensiled tomato peel	*Prevotellaceae*_Uncultured Genus-004;	✧ Increased microbial diversity in the gastrointestinal tract and increased ash digestibility	Buffalo	[32]
Cassava residue and dried distiller’s grain	Reduced *Prevotella* richness	✧ Significant increase in average daily weight gain and improved growth performance	Buffalo	[101]
Corn gluten and palm kernel cake	Increased *Prevotella* load	✧ Reduced average daily weight gain and digestibility	Buffalo	[101]
The substitution of 33% corn starch with barley starch in the diet	*Prevotella brevis*	✧ Improved rumen environment and feed efficiency	Sheep	[102]
Fermented total mixed ration containing cottonseed meal	*Prevotella*	✧ Increased volatile fatty acids yield, increased average daily weight gain, and promoted the performance	Sheep	[103]
Thymol Supplementation	Prevotella melaninogenica; Prevotella ruminicola	✧ Detrimental to the breakdown of proteins and fibrous material	Goats	[42]
Indoor feeding regimes	*Prevotella*	✧ Regulate amino acids, lipid and carbohydrate metabolism in muscle tissues and improve meat quality	Tibetan sheep	[105]
Yeast polysaccharide	*Prevotella*	✧ The metabolic mechanisms responsible for the growth-promoting and immune-regulating effects	Donkeys	[15]
Corn straw diets were fed ad libitum in addition to a commercial concentrate diet	*Prevotella*	✧ Involved in lipid metabolism and immune response	Donkeys	[86]
Tryptophan, Glutamate	Prevotellaceae-NK3B31 group, and UCG-005	✧ Improve gut health by stabilizing the intestinal environment and immune state	Pigs	[34]
Vanillic acid	Reduced *Prevotella* 9, *Prevotella* 7, *Prevotella* 2 and *Prevotella* 1 abundance	✧ Increased the final body weight and average daily gain	Piglets	[106]

## Data Availability

Data contained within the article.

## Abbreviations

GAA	Guanidinoacetic acid
LPSN	List of Prokaryotic names with Standing in Nomenclature
AAI	Average amino acid identity
POCP	Percentage of conserved proteins
Pfam	Pan-genomic identification of specific marker genes, protein families
CAZyme	Carbohydrate-activating enzyme
GI	Gastrointestinal
PULs	Polysaccharide Utilization Loci
SCFA	Short-chain fatty acids
